# Supplementary material for: Repeatedly experiencing the McGurk effect induces long-lasting changes in auditory speech perception
Source: Commun Psychol. 2024 Apr 3;2:25. doi: 10.1038/s44271-024-00073-w (PMC11332120; doi:10.1038/s44271-024-00073-w)
Supplement: Supplementary file 2 — Reporting Summary [file 44271_2024_73_MOESM2_ESM.pdf]

nature portfolio

Corresponding author(s): Michael Beauchamp (beaucha@upenn.edu)

Last updated by author(s): Feb 20, 2024

Reporting Summary

Nature Portfolio wishes to improve the reproducibility of the work that we publish. This form provides structure for consistency and transparency in reporting. For further information on Nature Portfolio policies, see our Editorial Policies and the Editorial Policy Checklist.

Statistics

For all statistical analyses, confirm that the following items are present in the figure legend, table legend, main text, or Methods section.

n/a	Confirmed	
☐	☒	The exact sample size (*n*) for each experimental group/condition, given as a discrete number and unit of measurement
☐	☒	A statement on whether measurements were taken from distinct samples or whether the same sample was measured repeatedly
☐	☒	The statistical test(s) used AND whether they are one- or two-sided<br>*Only common tests should be described solely by name; describe more complex techniques in the Methods section.*
☐	☒	A description of all covariates tested
☐	☒	A description of any assumptions or corrections, such as tests of normality and adjustment for multiple comparisons
☐	☒	A full description of the statistical parameters including central tendency (e.g. means) or other basic estimates (e.g. regression coefficient) AND variation (e.g. standard deviation) or associated estimates of uncertainty (e.g. confidence intervals)
☐	☒	For null hypothesis testing, the test statistic (e.g. *F*, *t*, *r*) with confidence intervals, effect sizes, degrees of freedom and *P* value noted<br>*Give P values as exact values whenever suitable.*
☐	☒	For Bayesian analysis, information on the choice of priors and Markov chain Monte Carlo settings
☐	☒	For hierarchical and complex designs, identification of the appropriate level for tests and full reporting of outcomes
☐	☒	Estimates of effect sizes (e.g. Cohen's *d*, Pearson's *r*), indicating how they were calculated

Our web collection on statistics for biologists contains articles on many of the points above.

Software and code

Policy information about availability of computer code

Data collection	Data was collected using online data collection services.
Data analysis	All data analysis code may be found in the file ReproducibleResults

For manuscripts utilizing custom algorithms or software that are central to the research but not yet described in published literature, software must be made available to editors and reviewers. We strongly encourage code deposition in a community repository (e.g. GitHub). See the Nature Portfolio guidelines for submitting code & software for further information.

Data

Policy information about availability of data

All manuscripts must include a data availability statement. This statement should provide the following information, where applicable:

- Accession codes, unique identifiers, or web links for publicly available datasets
- A description of any restrictions on data availability
- For clinical datasets or third party data, please ensure that the statement adheres to our policy

All data may be found in the file SummaryData.xlsx.

Human research participants

Policy information about studies involving human research participants and Sex and Gender in Research.

Reporting on sex and gender	Participants self-identified as "Male ", "Female" or "Other".
Population characteristics	Mean age was 26 years (range 19 - 43). Participants self-identified as "Male" (n = 17), "Female" (n = 11) or "Other" (n = 0). Data on race and ethnicity was not collected. All participants affirmed that they did not have a hearing impairment that would make it difficult to understand words; that they did not have an uncorrected visual impairment that would make it difficult to watch a video of a person talking; and that they were able to either complete tasks in a quiet environment or use noise-cancelling headphones.
Recruitment	Participants were recruited online
Ethics oversight	All experiments were approved by the Institutional Review Board of the University of Pennsylvania.

Note that full information on the approval of the study protocol must also be provided in the manuscript.

Field-specific reporting

Please select the one below that is the best fit for your research. If you are not sure, read the appropriate sections before making your selection.

☐ Life sciences ☒ Behavioural & social sciences ☐ Ecological, evolutionary & environmental sciences

For a reference copy of the document with all sections, see nature.com/documents/nr-reporting-summary-flat.pdf

Behavioural & social sciences study design

All studies must disclose on these points even when the disclosure is negative.

Study description	The study examined human speech perception. Participants reported their perception of a variety of auditory and audiovisual speech. The data was analyzed quatitatively.
Research sample	The research sample consisted of participants tested online. Mean age was 26 years (range 19 - 43).
Sampling strategy	To estimate the necessary sample size for detecting changes in auditory perception, we assumed that fusion responses to auditory-only stimuli in the pre-test would occur (due to errors in perception or response selection) less than 1% of the time, and that changed auditory perception would occur on at least 20% of trials in the post-test. We estimated (using G*Power, McNemar test for inequality of dependent proportions) that a sample size of 25 would produce 80% power to detect a 25-fold increase in the odds of a fusion response to an auditory-only stimulus (alpha = 0.05, one-tailed; proportion discordant pairs = 0.3).
Data collection	All data was collected online from participants using online testing services.
Timing	For the main experiment, data collection started on March 23 2022 and ended on April 7 2022.<br>For the replication experiment, data collection started on August 13 2022 and ended on August 29 2022.
Data exclusions	For the main experiment, thirty participants completed the pre-test. Two participants performed poorly and were excluded from further analysis, leaving 28 participants. For the replication experiment, no participants were excluded.
Non-participation	No participants dropped out.
Randomization	Participants were not allocated into different experimental groups.

Reporting for specific materials, systems and methods

We require information from authors about some types of materials, experimental systems and methods used in many studies. Here, indicate whether each material, system or method listed is relevant to your study. If you are not sure if a list item applies to your research, read the appropriate section before selecting a response.

Materials & experimental systems

n/a	Involved in the study
☒	☐ Antibodies
☒	☐ Eukaryotic cell lines
☒	☐ Palaeontology and archaeology
☒	☐ Animals and other organisms
☒	☐ Clinical data
☒	☐ Dual use research of concern

Methods

n/a	Involved in the study
☒	☐ ChIP-seq
☒	☐ Flow cytometry
☒	☐ MRI-based neuroimaging
